# Padlock and Proximity Probes for *in Situ* and Array-Based Analyses: Tools for the Post-Genomic Era

**DOI:** 10.1002/cfg.326

**Published:** 2003-10

**Authors:** Ulf Landegren, Fredrik Dahl, Mats Nilsson, Simon Fredriksson, Johan Banér, Mats Gullberg, Jonas Jarvius, Sigrun Gustafsdottir, Ola Söderberg, Olle Ericsson, Johan Stenberg, Edith Schallmeiner

**Affiliations:** Department of Genetics and Pathology Rudbeck Laboratory Uppsala University Sweden

## Abstract

Highly specific high-throughput assays will be required to take full advantage of the accumulating information about the macromolecular composition of cells and tissues, in order to characterize biological systems in health and disease. We discuss the general problem of detection specificity and present the approach our group has taken, involving the reformatting of analogue biological information to digital reporter segments of genetic information via a series of DNA ligation assays. The assays enable extensive, coordinated analyses of the numbers and locations of genes, transcripts and protein.


**Comparative and Functional Genomics**
*Comp Funct Genom* 2003; **4**: 525–530.
Published online in Wiley InterScience (www.interscience.wiley.com). **DOI:** 10.1002/cfg.326

**Conference Review**

### Padlock and proximity probes for *in situ* and array-based analyses: tools for the post-genomic era

Ulf Landegren*, Fredrik Dahl, Mats Nilsson, Simon Fredriksson, Johan Banér, Mats Gullberg, Jonas Jarvius, Sigrun Gustafsdottir, Ola Söderberg, Olle Ericsson, Johan Stenberg and Edith Schallmeiner
*Department of Genetics and Pathology, Rudbeck Laboratory, Uppsala University, Sweden*

**Correspondence to:*
*Ulf Landegren, Department of Genetics and Pathology, Rudbeck Laboratory, Uppsala University, Sweden.*
*E-mail:*
*ulf.landegren@genpat.uu.se*

Received: 6 June 2003
Revised: 5 August 2003
Accepted: 5 August 2003

### Abstract

**Highly specific high-throughput assays will be required to take full advantage of the accumulating information about the macromolecular composition of cells and tissues, in order to characterize biological systems in health and disease. We discuss the general problem of detection specificity and present the approach our group has taken, involving the reformatting of analogue biological information to digital reporter segments of genetic information via a series of DNA ligation assays. The assays enable extensive, coordinated analyses of the numbers and locations of genes, transcripts and protein. Copyright © 2003 John Wiley & Sons, Ltd.**

### A background to molecular analyses

Complete genome sequences are becoming available for an increasing number of organisms, allowing research on the corresponding species to transit to a post-genomic phase. Studies can now be founded on extensive parts lists, comprising all protein-coding genes as well as regulatory and structural genetic elements, common genetic variants and predicted repertoires of proteins of these organisms. It remains contentious just how sense can best be made of variable patterns of gene expression, of skewed distribution of common genetic variants among healthy and affected individuals, or of the representation of protein sets in different samples. Nonetheless, the opportunity to specifically demonstrate the presence, concentration, distribution and relative location of all these molecular components clearly will provide a basis for entirely new insights into normal biological processes and disease mechanisms.

Despite impressive progress in development of tools for macromolecular analysis, such techniques remain crucial limiting factors for capitalizing on genomic information in biological studies. At its core, the problem of analysing macromolecules in biological samples is one of specificity of detection, and the requirements are extreme: the two double-stranded haploid genomes in any human interphase cell together comprise approximately 13 billion nucleotides that must be searched to find a particular single-nucleotide variant. An unexpectedly large proportion of the genome, maybe half, can be transcribed to RNA (Scherer *et al.*, 2003), but here the detection problem is compounded by the need to distinguish related sequences that may be present at vastly different copy numbers. This same problem is still more pressing at the level of protein and, in addition, subtle modifications of the proteins must be distinguished as these can signal entirely different activity states. The number of functionally distinct protein species that need be resolved in an organism remains largely unknown. Finally, in order to understand cellular functions it is also important to obtain information about the organization of all these molecules on length scales between tens of nanometers to micrometers and above. It is necessary to clarify the architecture of interacting molecules and to chart sub-ecologies within cells, as well as relationships between cells in tissues.

Copyright © 2003 John Wiley & Sons, Ltd.

Highly precise detection mechanisms are thus required to convert the analogue information embodied in the states of cells to digital information about the concentration and distribution of cellular components. While methods like PCR provide the specificity required to search highly complex genomes, only a limited number of target sequences can be investigated in individual reactions, and spatial information is typically lost. Conversely, DNA hybridization reactions provide localized information, and large numbers of targets can be investigated in parallel in array-based analyses, but due to inherent limitations in the specificity of hybridization it is generally not possible to investigate complex samples such as the total human genome, and detection of rare RNA sequences is difficult. The analogous problems exist for analyses of proteins: absolute specificity of protein binding is probably not attainable and assay performance therefore crucially depends on test architectures, most of which were established several decades ago. Just as pairs of primers are required to elicit PCR amplification, assays dependent on pairwise antibody binding provide increased specificity, but problems of cross-reactivity mount when large numbers of molecules are investigated in parallel and the mechanism currently is not applicable for localized detection.

In this review, we will discuss reaction mechanisms developed by our group for advanced macromolecular analyses. The three main interrelated technologies to be described are padlock probes and proximity ligation probes for nucleic acid and protein analyses, respectively, and rolling-circle amplification as a general means of sensitive localized and solution-phase detection.

### Ligation-based techniques for nucleic acid analyses

An ideal set of molecular tools for macromolecular analysis would allow simple, automated design and synthesis of sets of reagents that can then be combined in large numbers, and in a seamless process result in detection signals that are easily recorded over background. The general strategy we have adopted, to be described in greater detail below, achieves these ends by reformatting biological information as sets of signature DNA sequences, created in target-dependent probe ligation reactions.

The possibility of analysing nucleic acid molecules according to their potential to serve as targets for DNA ligation reactions has a history that stems back to the work by Gobind Khorana's group in the early 1970s, in which a DNA sequence encoding a tRNA molecule was shown to template probe ligation reactions (Besmer *et al.*, 1972). In the late 1980s we and others established analytic DNA ligation in the oligonucleotide ligation assay (OLA), one of a small number of mechanisms still in use today for analysing specific target nucleic acid sequences (Landegren *et al.*, 1988; Alves and Carr, 1988).

In OLA, pairs of probes are joined, forming new DNA strands, if and only if the probe pairs are juxtaposed by hybridizing next to each other on the same target sequence, forming a substrate for DNA ligation. The requirement for coordinated hybridization by pairs of probes renders assays sufficiently specific to detect and distinguish single-copy gene sequences directly in total genomic DNA, and the enzyme's substrate requirements easily distinguish any DNA sequence differences leading to mismatched ligation substrates. However, just as with PCR, and with sandwich immunoassays for protein detection, the paired-probe design can give rise to cross-reactions, as more targets are investigated in the same reaction. We therefore developed so-called padlock probes — oligonucleotide probes designed so that target-complementary sequences at both ends can hybridize in juxtaposition on target strands and be joined by ligation in a target-dependent manner (Nilsson *et al.*, 1994; Figure 1). The two target-complementary segments of the probes are separated by sequences that can be used for amplification or identification via DNA tag sequences.

The padlock probe design offers a number of advantages, compared both to standard hybridization probes and to PCR and OLA. (a) The ligation reaction converts linear oligonucleotide probes to circular DNA strands, wound around target molecules. This means that probes specifically bound to long target molecules can withstand washes under conditions where no base-pairing persists, i.e. at denaturing pH or temperature, without detaching. (b) It is also possible to apply exonuclease enzymes that degrade nucleic acids from the 5′ and/or 3′ ends in order to remove any unreacted probes, while preserving reacted — endless — probes. (c) Finally, as will

Copyright © 2003 John Wiley & Sons, Ltd. 

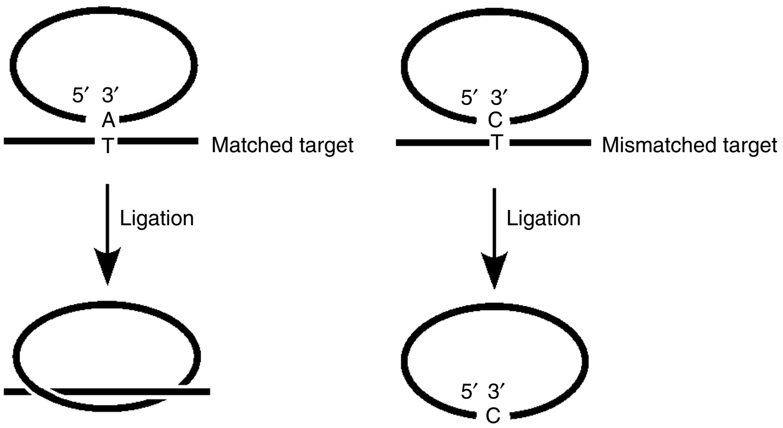

**Figure 1.** Padlock probe ligation for nucleic acid detection. Oligonucleotide probes with end sequences correctly hybridized in juxtaposition on a target strand can be ligated to form circular DNA strands, wound around the target sequence. By contrast, probes mismatched at the 3′ position are poor substrates for ligation, allowing sequence variants to be distinguished be discussed below, circularized probe molecules may be copied in a rolling-circle replication reaction, reeling off a long, single-stranded molecule that represents catenated repeats of the complement of the ligation probe.

Any intermolecular ligation products (that may arise through cross-reaction) fail to encircle the target, or to template rolling-circle replication, and can be digested with exonucleases. All these properties can be exploited to remove any cross-reactive products while preserving specifically reacted probes, thus enabling highly parallel assays. In collaboration with the group of Ron Davis, we have demonstrated that large numbers of padlock probes may be added to a genomic DNA sample to interrogate more than 1000 single nucleotide polymorphisms (SNPs) (Hardenbol *et al.*, 2003). After target-dependent circularization and exonuclease treatment, all reacted probes are amplified in a single amplification reaction. Amplification products representing individual loci are identified by hybridizing to a general tag microarray via locus-specific signature sequences. Since submitting this paper, the company ParAllele BioScience has demonstrated that the method allows analyses of sets of 13 000 SNPs per reaction.

RNA molecules have been known to template ligation of DNA oligonucleotides hybridizing in tandem to RNA molecules since ligases were first investigated in the 1960s. We have optimized conditions for detection of RNA target sequences via oligonucleotide ligation reactions (Nilsson *et al.*, 2000, 2001). As an alternative, we have also used parallel padlock analyses to measure the relative representation of allelic transcripts at the level of cDNA (Banér *et al.*, 2003). It will now be important to target other limiting factors for further increases in the numbers of target DNA or RNA molecules that can be analysed in parallel using padlock probes. Accordingly, we have developed a computer program to assist in the design of large sets of padlock probes (Stenberg *et al.*, in preparation), and we are investigating improved means of synthesizing large sets of probes.

### Ligation-based protein analysis

We have recently demonstrated that the detection of proteins can also be monitored via coupled DNA ligation assays. The ligation reactions generate amplifiable signature sequences that represent the analysed proteins. This so-called 'proximity ligation procedure' uses specific protein binding reagents with attached oligonucleotides. When pairs of reagents bind a target protein molecule, then the DNA strands can be joined by ligation in a proximity-dependent reaction, followed by amplification by PCR of the newly formed DNA strand with real-time detection (Fredriksson *et al.*, 2002; Figure 2). The assay mechanism can be applied in a homogenous assay format, with no need for washes, for the detection of amounts of platelet-derived growth factor 1000 times lower than those

Copyright © 2003 John Wiley & Sons, Ltd. 

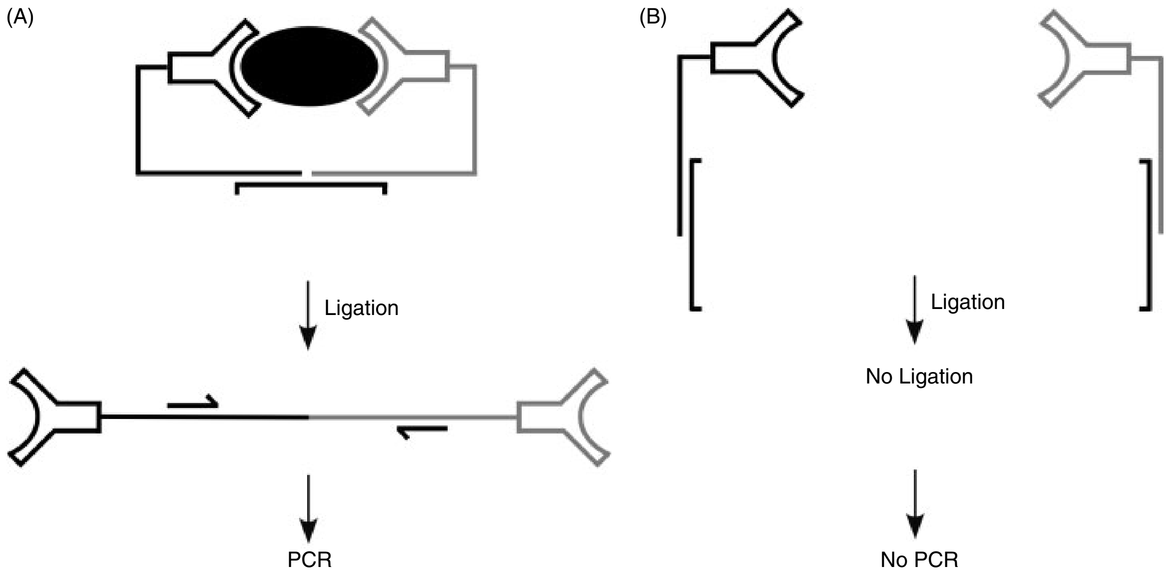

**Figure 2.** Proximity ligation for protein detection. (A) Binding of a pair of protein-binding reagents with attached DNA strands to a target protein brings the DNA strands into proximity. Next, a connector oligonucleotide is added in excess, allowing DNA ends that have been brought close to be joined by ligation, forming a substrate for amplified detection, e.g. via real-time PCR. (B) DNA strands on binding reagents that have failed to be brought close by binding in pairs to a target protein hybridize to one connector oligonucleotide each. Thereby the strands become unable to undergo subsequent ligation and amplification detected by solid-phase ELISA. Very small samples can be analysed and low concentrations of reagents are required. We have also described a solid support-based form of the assay in which target protein molecules, captured on a solid support via antibodies, were exposed to pairs of proximity probes. After washes, the strands were joined by ligation, followed by amplified detection of the DNA strand that formed. This version of the assay yields even greater specificity of detection, as it requires three specific binding events and it allowed detection of still lower concentrations of target proteins. Proximity ligation assays should also be suitable to monitor numerous proteins in parallel, by representing these as distinct tag DNA sequences. The same proximity-dependent mechanism can also be used for specific localized detection of target proteins, as discussed below.

The first protein binding reagents we used in the proximity ligation assay were so-called 'DNA aptamers', equipped with ligatable DNA sequence extensions to serve as proximity probes. DNA aptamers are stable to storage and they can easily be modified with DNA sequence extensions. Furthermore, they can be reproduced in different laboratories through standard oligonucleotide synthesis, on the basis of published nucleotide sequence information. Relatively few suitable aptamers have been described in the literature, however, and more recently we have established protocols to convert other classes of binding reagents, such as mono- and polyclonal antibodies to proximity probes, establishing the mechanism as a promising, quite general approach for protein analyses (Gullberg *et al.*, in preparation).

### Rolling-circle replication for sensitive parallel and localized assays

Both the detection of target DNA and RNA sequences by padlock probes, as well as protein detection by proximity probes, can be reflected in the formation of circular DNA strands. We and others have demonstrated that circular probes are substrates for a form of DNA amplification known as rolling-circle replication (Fire and Xu, 1995; Kool, 1996; Baner *et al.*, 1998; Lizardi *et al.*, 1998). Using an appropriate DNA polymerase, the rolling-circle replication reaction can yield a single-stranded DNA molecule, composed of 1000

Copyright © 2003 John Wiley & Sons, Ltd. 

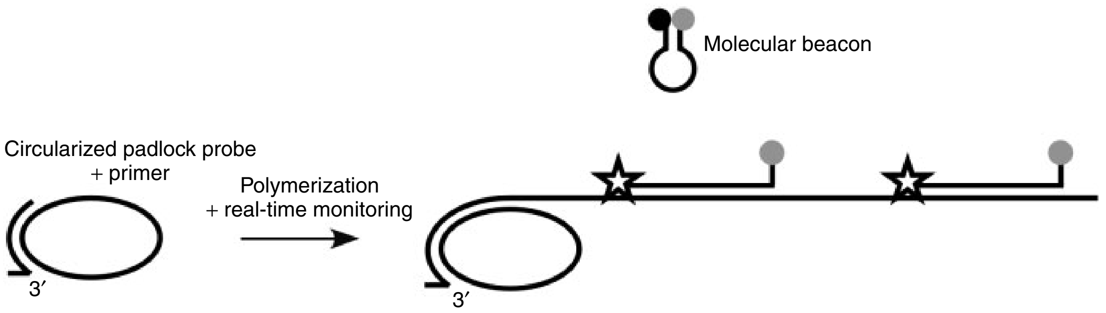

**Figure 3.** Rolling-circle replication for signal amplification. Circularized probes can act as templates for rolling-circle replication, resulting in a single-stranded product composed of repeated complements of the DNA circle. The polymerization can be monitored in real time with excellent quantitative resolution, using strand-specific molecular beacons that hybridize to the product complements of the circularized probe, all strung together. The progress of amplification can be monitored in real time using modified molecular beacons to probe the amplification products, resulting in highly accurate measurement of the numbers of starting DNA circles (Nilsson *et al.*, 2002; Figure 3). We have furthermore found that it is possible to use a modified protocol in order to amplify the circularized probes in a controlled manner, producing a billion-fold increase of DNA circles in the course of a few hours (Dahl *et al.*, in preparation).

It is of particular value that the rolling-circle replication mechanism can also be used to elicit strong localized detection reactions, reporting the location of target DNA and RNA molecules that have been specifically detected by padlock probes (Nilsson *et al.*, in preparation). In the same manner it is also possible to initiate rolling-circle replication reactions from the pairwise binding of antibodies to localized target proteins for highly sensitive and specific localized detection of molecules (Gullberg *et al.*, in preparation). The combination of highly specific target-detection using padlock and proximity ligation probes, giving rise to circular DNA strands that carry sequence elements identifying the recognized molecules, allows for an integral chain of specificity from target recognition to visualization of rolling-circle replication products.

### Conclusions

Molecular tools are, or will become, available that allow standardized analyses of very large sets of target nucleic acids and proteins, without undue attention to probe construction or assay parameters for particular target sets. Equally, it will be possible to analyse very large numbers of samples in parallel using advanced probing strategies. Ligation reactions are promising as means of reformatting a bewildering array of DNA, RNA and protein analytes to signature DNA sequences, through a uniform set of automated and miniaturized assay steps. Detection by specific probes inexorably leads to the formation of circular DNA molecules that, in turn, yield detection signals amplified far above background noise, and with minimal opportunities for false-positive reactions along the way. The probing strategies presented herein also promise to blend analyses of all classes of macromolecules into common assay formats, allowing parallel analyses. Moreover, the very same specific probes prepared for high-throughput solution-phase assays can equally well be applied to the detection of the location, and the spatial relations, of these same target molecules. Accordingly, DNA ligation-based assays are promising as general post-genomic analytical tools.

### Acknowledgements

Work in our group is supported by the Beijer and Wallenberg Foundations, by Polysaccharide Research AB (Uppsala) and by the Research Councils of Sweden for Natural Science and for Medicine.

### References

Alves AM, Carr FJ. 1988. Dot blot detection of point mutations with adjacently hybridising synthetic oligonucleotide probes. *Nucleic Acids Res* **16**: 8723.

Copyright © 2003 John Wiley & Sons, Ltd. 

Banér J, Nilsson M, Mendel-Hartvig M, Landegren U. 1998. Signal amplification of padlock probes by rolling circle replication. *Nucleic Acids Res* **26**: 5073–5078.

Banér J, Isaksson A, Waldenström E, *et al*. 2003. Parallel gene analysis with allele-specific padlock probes and tag microarrays. *Nucleic Acids Res* **31**: 103.

Besmer P, Miller RC Jr, Caruthers, *et al*. 1972. Studies on polynucleotides. CXVII. Hybridization of polydeoxynucleotides with tyrosine transfer RNA sequences to the r-strand of phi80psu + 3 DNA. *J Mol Biol* **72**: 503–522.

Fire A, Xu SQ. 1995. Rolling replication of short DNA circles. *Proc Natl Acad Sci USA* **92**: 4641–4645.

Fredriksson S, Gullberg M, Jarvius J, *et al*. 2002. Protein detection using proximity-dependent DNA ligation assays. *Nature Biotechnol* **20**: 473–477.

Hardenbol P, Baner J, Jain M, *et al*. 2003. Multiplexed genotyping with sequence-tagged molecular inversion probes. *Nature Biotechnol* **21**: 673–678.

Kool ET. 1996. Circular oligonucleotides: new concepts in oligonucleotide design. *Ann Rev Biophys Biomol Struct* **25**: 1–28.

Landegren U, Kaiser R, Sanders J, Hood L. 1988. A ligase-mediated gene detection technique. *Science* **241**: 1077–1080.

Lizardi PM, Huang X, Zhu Z, *et al*. 1998. Mutation detection and single-molecule counting using isothermal rolling-circle amplification. *Nature Genet* **19**: 225–232.

Nilsson M, Antson DO, Barbany G, Landegren U. 2001. RNA-templated DNA ligation for transcript analysis. *Nucleic Acids Res* **29**: 578–581.

Nilsson M, Barbany G, Antson DO, Gertow K, Landegren U. 2000. Enhanced detection and distinction of RNA by enzymatic probe ligation. *Nature Biotechnol* **18**: 791–793.

Nilsson M, Gullberg M, Dahl F, Szuhai K, Raap AK. 2002. Real-time monitoring of rolling-circle amplification using a modified molecular beacon design. *Nucleic Acids Res* **30**: e66.

Nilsson M, Malmgren H, Samiotaki M, *et al*. 1994. Padlock probes: circularizing oligonucleotides for localized DNA detection. *Science* **265**: 2085–2088.

Scherer SW, Cheung J, MacDonald JR, *et al*. 2003. Human chromosome 7: DNA sequence and biology. *Science* **300**: 767–772.

Copyright © 2003 John Wiley & Sons, Ltd. 
